# Identification of *Spiropoeus fischeri* Brandt, 1833 from captive bred millipedes in Zoos (Diplopoda, Spirostreptida, Spirostreptidae)

**DOI:** 10.3897/BDJ.14.e196645

**Published:** 2026-08-25

**Authors:** Santiago Alvear, Carter J. Young, Luisa F. Vasquez-Valverde, Paul E. Marek

**Affiliations:** 1 Virginia Tech, Department of Entomology, Blacksburg, United States of America Virginia Tech, Department of Entomology Blacksburg United States of America

**Keywords:** Benin, Cameroon, Congo, giant African millipede, gonopod, Liberia, Spirostreptini

## Abstract

**Background:**

Large millipedes of the family Spirostreptidae are often kept in zoos and other educational living collections in the United States where they are commonly known as giant African millipedes. Although accurate species determinations are needed for permitting, live animal care protocols and outreach, many of these millipedes originate from poorly-documented sources (e.g. the exotic species trade). This leaves species determination unclear and hampers understanding and communicating the species natural history and other biological information.

**New information:**

We found that *Spiropoeus fischeri* Brandt, 1833 is the identity of a large millipede commonly exhibited alive by zoos in the U.S. Although this large (14 cm-long), easy-to-raise millipede has been kept for decades in living exhibits, its identity was unknown until now. We provide a detailed explanation of the diagnostic morphology to identify *S. fischeri*, and supply cytochrome oxidase subunit I (COI) DNA barcode sequences of individuals. With accurate knowledge of its identity, zoos and educational collections can now share with the public a species name and rich biological information about *S. fischeri*, including its life history and home range in sub-Saharan Africa.

## Introduction

Large tropical millipedes are commonly kept alive in zoos and other educational collections in the United States. The provenance of these animals is varied and unclear, but many of them were obtained from the exotic pet trade or other ambiguous sources. Most of these millipedes belong to African species of the family Spirostreptidae and are colloquially known as giant African millipedes. Their large body size, gentle nature and well-established husbandry techniques make them ideal for outreach (Sigling 2010). Despite their immense value for education and engagement of the public with nature and biodiversity, the species identity of captive millipedes is often uncertain. This is due to poor record-keeping and limited taxonomic expertise of practitioners of the commercial invertebrate pet trade — often the source of the animals. Difficulty with identification is exacerbated by the challenge of obtaining and understanding taxonomic literature and tracing specimens back to their original distributions. An accurate species determination is necessitated for animal care guidebooks and permits for their trade amongst zoos such as the U.S. Fish and Wildlife Service Declaration form 3-177. Additionally, biological information about the species, such as its distribution and habitat, would enhance the public’s educational experience.

African Spirostreptidae include several notable species, such as *Archispirostreptus gigas* (Peters, 1855), which is the longest millipede in total body length (up to 30 cm) and *Archispirostreptus syriacus* (DeSaussure, 1859) — the millipede with the longest lifespan at 9 years (Crawford et al. 1986, Hopkin and Read 1992). The internal classification of the Spirostreptidae Pocock, 1894 has been historically unstable. Some authors have attempted to divide the family into subfamilies or tribes, but none of these classifications have been widely accepted and the relationships below family level to date remain unresolved (Krabbe 1982, Enghoff 2023). Although the Spirostreptidae of eastern Africa have received more attention in recent years (Enghoff et al. 2024), West African genera generally have remained understudied, but see Mwabvu et al. (2010).

The monotypic genus *Spiropoeus* Brandt, 1833, with its only species *Spiropoeus fischeri* Brandt, 1833, was described from material collected from West Africa, although the precise locality is unknown. The type locality of *Spiropoeus* and *Spirostreptus*, both described by Brandt (1833), are unclear, but were part of the renowned Seba Cabinet of the Kunstkamera of Tsar Peter I (Golovatch and Hoffman 2000, Hoffman et al. 2001). Several West African species and subspecies originally described in the genera *Spirostreptus* Brandt, 1833 and *Mardonius* Attems, 1914 have been synonymised with *S. fischeri* following the revision of the genus by Hoffman et al. (2001). This suggests that *S. fischeri* is a widespread, morphologically variable and vaguely delineated species. Here, we provide an accurate determination of *Spiropoeus fischeri* from material obtained from zoos in the United States and outline its taxonomic history, distribution in the wild, diagnostic morphological characters and COI barcodes.

## Materials and methods

We examined two male specimens of large spirostreptid millipedes from the Akron Zoo (VTEC, MPE05526) and Omaha Zoo (VTEC, MPE05527). Specimens were deposited in the Virginia Tech Insect Collection (abbreviated VTEC). The gonopods were dissected according to Bond et al. (2003) and Means et al. (2015) and photographed with a Canon EOS 5D dSLR camera with a 65 mm Canon MP-E lens. The general morphology was viewed with a Leica M125 stereomicroscope.

To establish an expertly identified voucher of *S. fischeri* for DNA and to support future identification, we extracted and amplified the COI gene from both specimens. DNA extraction, PCR and sequencing was carried out according to the methods in Means and Marek (2017). The two sequences are identical to one another. No COI sequences of *S. fischeri* were available in public databases. A BLAST search in the National Center for Biotechnology Information (NCBI) returned relatively low identity matches (~ 83%) to sequences from the genus *Tropostreptus*, a spirostreptid genus from Tanzania (Enghoff 2017) and *Heptischius*, a Southeast Asian genus in the family Harpagophoridae (Pimvichai et al. 2010). An additional search in the Barcode of Life Data System (BOLD) yielded a higher identity match (~ 95%) to a sequence from Gabon identified as a species of the family Nemasomatidae (order Julida). Given the distribution of the family Nemasomatidae, this voucher specimen is likely misidentified.

The COI DNA sequence from the voucher specimen of *S. fischeri* from the Akron Zoo (MPE05526) is accessioned at NCBI with the accession number PX681997 and a second *S. fischeri* specimen from the Omaha Zoo (MPE05527) has the accession number PX681998. Genomic DNA of MPE05526 and MPE05527 is held in the VTEC -80°C freezer collection.

## Taxon treatments

### Spiropoeus fischeri

Brandt, 1833

0DA76867-97EC-57C8-9127-646C0D9778F0

Spiropoeus fischeri Brandt, 1833 - Brandt (1833): 205. Type species *Spiropoeus fischeri* Brandt, 1833, by monotypy.
Spiropoeus fischeri Brandt 1833: 205, pl. 5, figs. 37–39; Gervais (1837): 47; Krabbe (1982): 32, fig. 9b; Golovatch and Hoffman (2000): 245, figs. 17–25; Hoffman et al. (2001): 43, figs. 8–11.
Julus (Spiropoeus) fischeri — Brandt (1841): 125.
Spirostreptus (Nodopyge) parilis Karsch 1881: 36. Synonymised by Hoffman et al. (2001): 43.
Spirostreptus (Nodopyge) lingulatus Karsch 1881: 45. Synonymised by Hoffman et al. (2001): 43.
*Spirostreptus liber Porat 1888*: 30. Synonymised by Hoffman et al. (2001): 43.
Scaphiostreptus parilis — Attems (1914): 87, pl. 3, figs. 66–67; Demange (1970): 390.
Scaphiostreptus parilis acuticonus Attems 1914: 88, pl. 3, fig. 68. Synonymised by Hoffman et al. (2001): 43.
*Scaphiostreptus parilis sternalis Brölemann 1926*: 56, figs. 46–49. Synonymised by Hoffman et al. (2001): 43.
Epistreptus (Epistreptus) parilis — Attems (1950): 189; Demange (1964): 198, figs. 10–13.
Mardonius parilis — Demange and Mauriès (1975): 40, figs. 62–63; Krabbe (1982): 428; Golovatch (1997): 99, figs. 19–21.
Mardonius parilis acuticonus — Demange (1971): 77; Krabbe (1982): 429.
Mardonius parilis sternalis — Krabbe (1982): 429.
Mardonius piceus — Attems (1952): 280, figs. 1–3; Demange (1964): 200.

#### Description

##### Material examined

**Figure 1.**
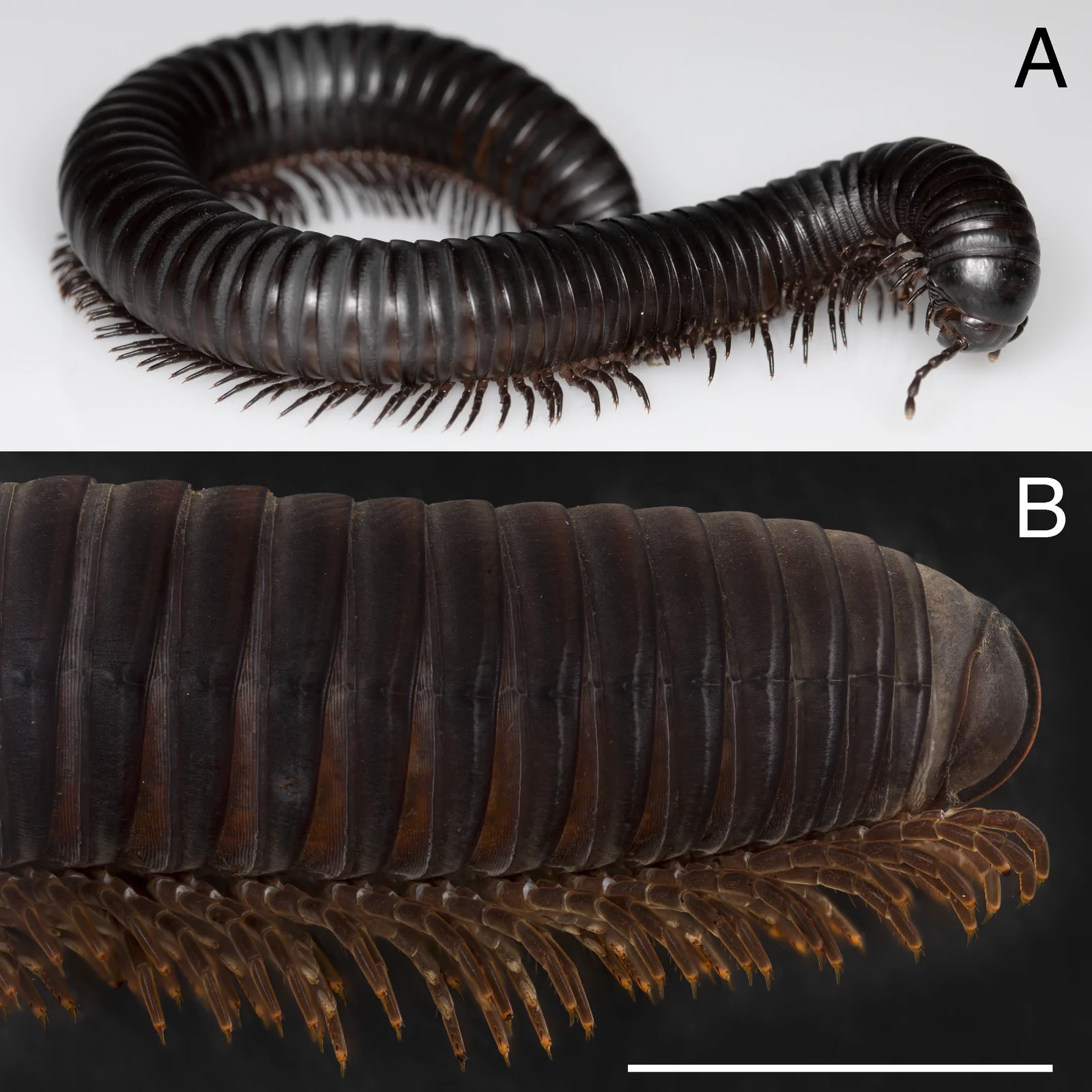
*Spiropoeus fischeri* Brandt, 1833 (MPE05527). **A** Habitus, live specimen; **B** Terminal body rings, lateral view. Scale bar: 10 mm.

**Figure 2.**
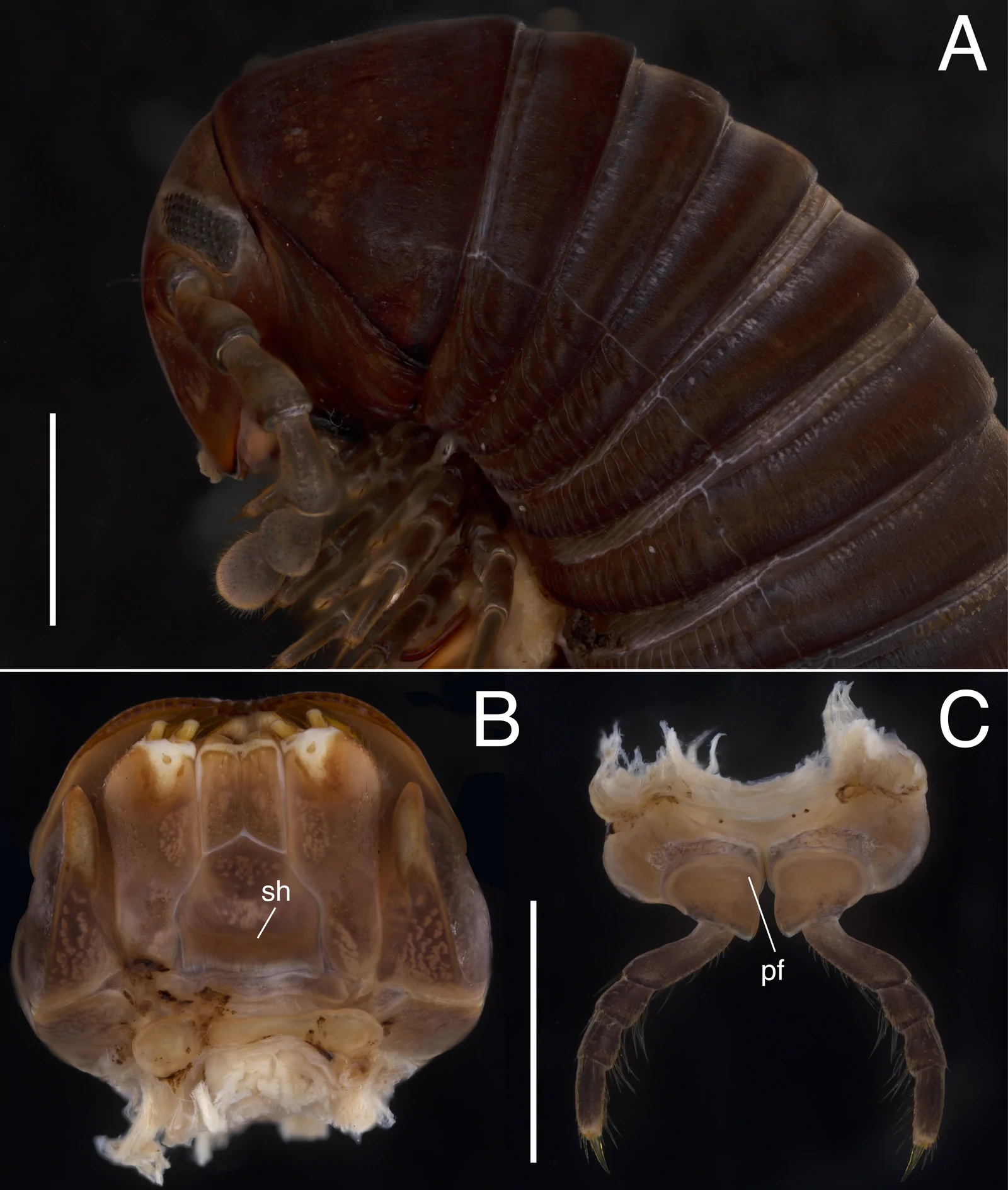
*Spiropoeus fischeri* Brandt, 1833 (A. MPE05527; B–C. MPE05526). **A** Head and anteriormost segments, lateral view; **B** Gnathochilarium, ventral view; **C** First leg pair, anterior view. Note the lack of a conical prefemoral process as in Hoffman et al. (2001). Abbreviations: shelf (**sh**); prefemur (**pf**).

**Figure 3.**
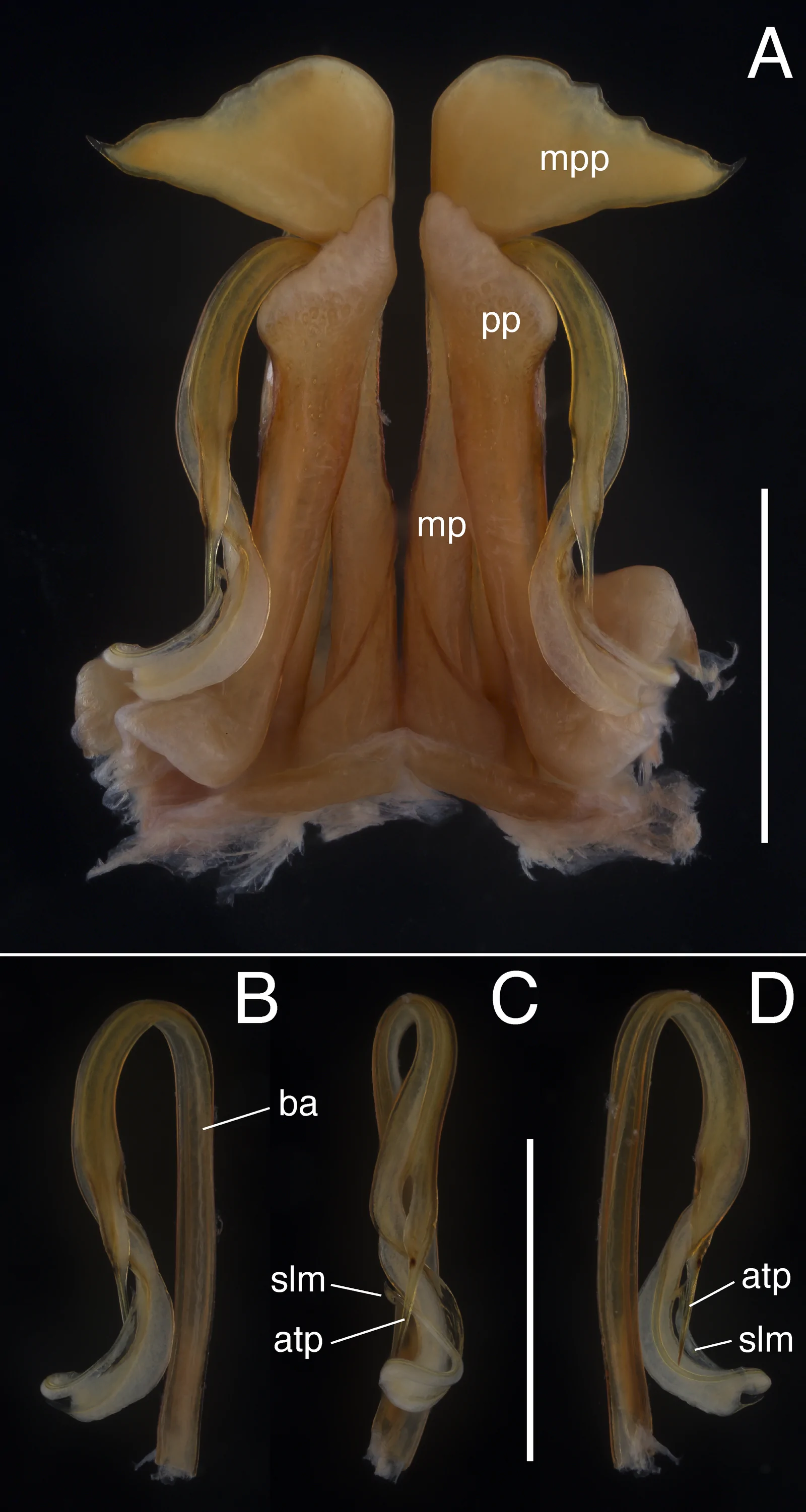
*Spiropoeus fischeri* Brandt, 1833 (MPE05526). **A** Gonopods, anterior view; **B** Left telopodite, anterior view; **C** Left telopodite, lateral view; **D** Left telopodite, posterior view. Scale bar: 2 mm. Abbreviations: antetorsal process (**atp**), basomere (**ba**), metaplica (**mp**), metaplical process (**mpp**), proplica (**pp**), solenomere (**slm**).

1♂, source location unknown, captive bred specimen from Akron Zoo, Akron, Ohio (MPE05526, VTEC); 1♂, source location unknown, captive bred specimen from Omaha's Henry Doorly Zoo and Aquarium, Omaha, Nebraska (MPE05527, VTEC) (Figs 1, 2, 3).

##### Rationale for determination as *S. fischeri*

A member of Spirostreptini, a tribe which possesses a terminally branched efferent (sperm) canal and with: (1) a metaplica bent 90° with triangular metaplical process (Fig. 3A, **mpp**); (2) apically acuminate antetorsal process (Fig. 3C and D, **atp**); (3) long serpentine solenomere (Fig. 3C and D, **slm**) and (4) with a projecting shelf on the mentum of the gnathochilarium (Fig. 2B, **sh**).

## Discussion

**Figure 4.**
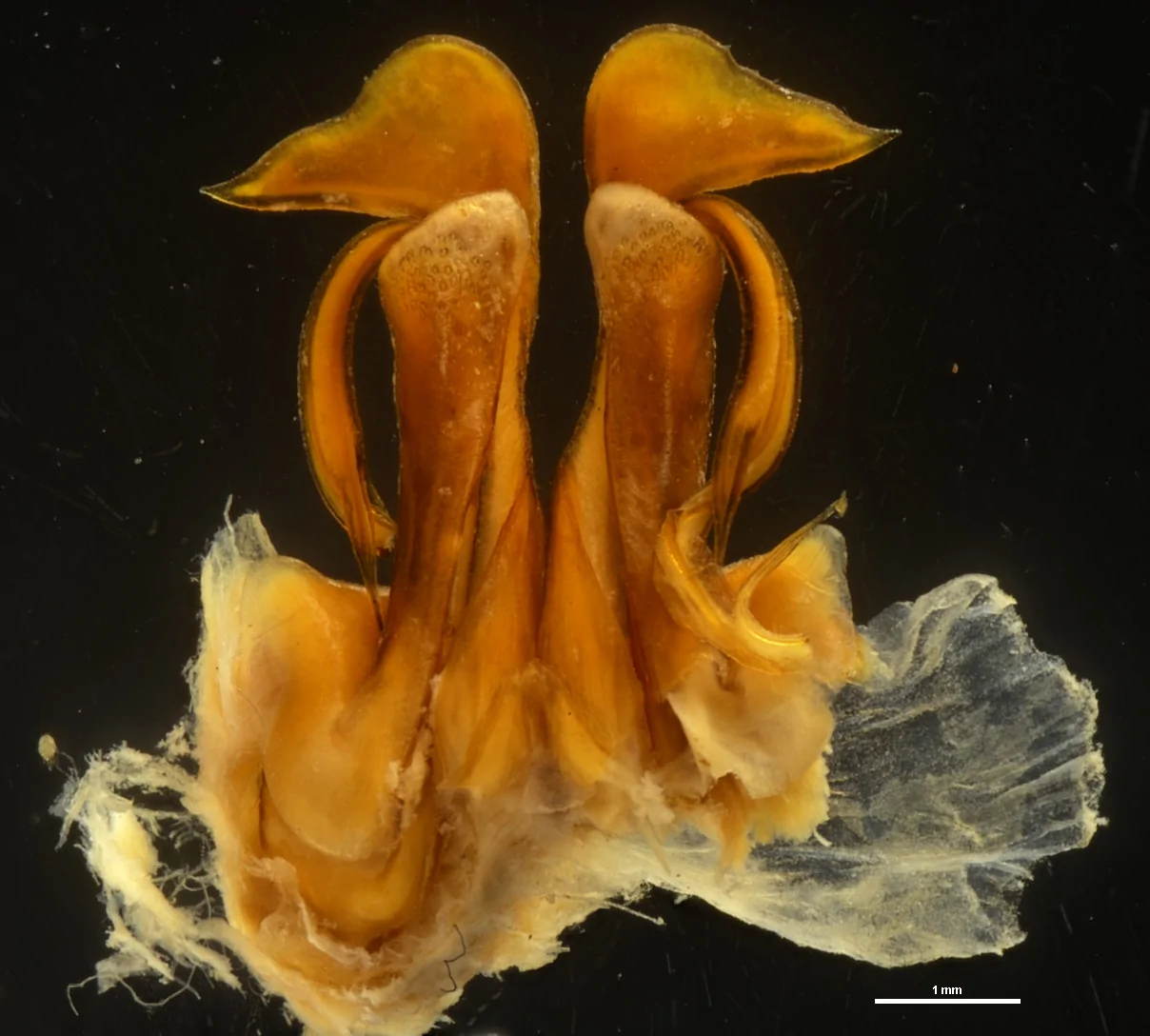
*Scaphiostreptus parilis acuticonus* Attems 1914 (= *Spiropoeus fischeri* Brandt, 1833). ♂ Holotype. Gonopods, anterior view. © Oliver Macek, 2025 Natural History Museum Vienna.

**Figure 5.**
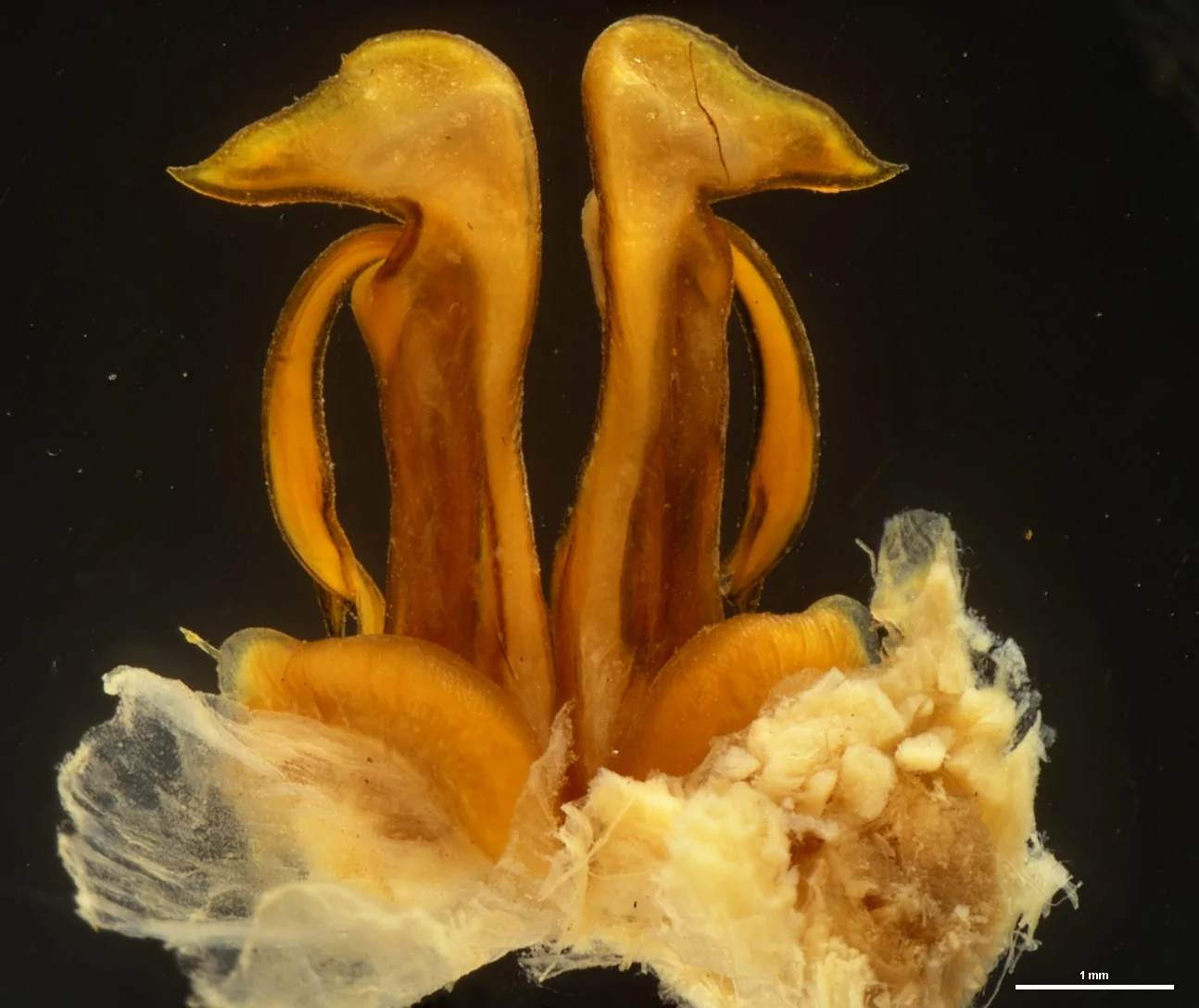
*Scaphiostreptus parilis acuticonus* Attems 1914 (= *Spiropoeus fischeri* Brandt, 1833). ♂ Holotype. Gonopods, posterior view. © Oliver Macek, 2025 Natural History Museum Vienna.

**Figure 6.**
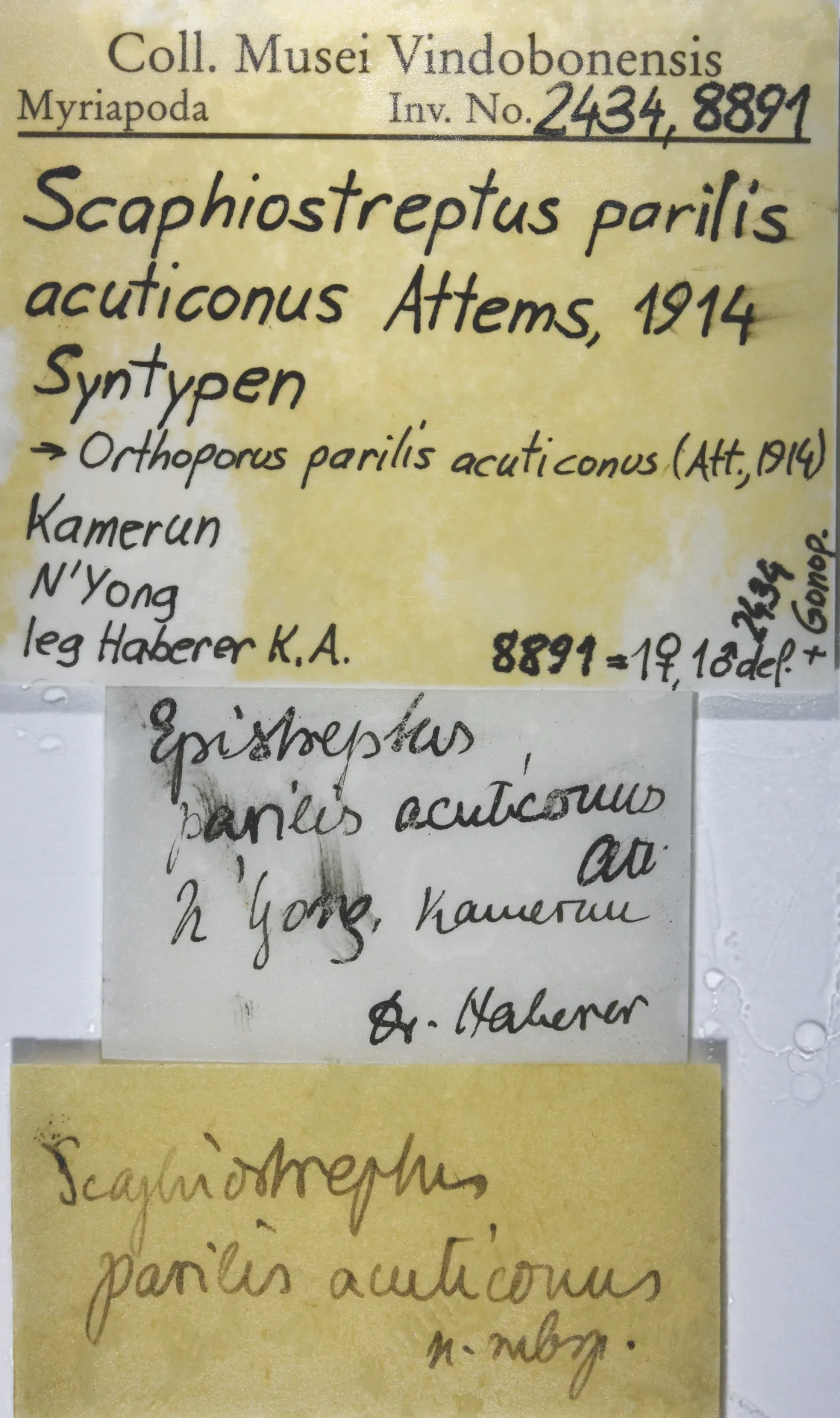
*Scaphiostreptus parilis acuticonus* Attems 1914 (= *Spiropoeus fischeri* Brandt, 1833). ♂ Holotype. Labels accompanying holotype with bottom label hand-written by Attems. © Oliver Macek, 2025 Natural History Museum Vienna.

We found that examined specimens from Akron and Omaha zoos match *Spiropoeus fischeri*. The overall body form, gonopod shape and configuration and the presence of a projecting shelf in the mentum of the gnathochilarium were consistent with previous illustrations and descriptions of the species (*Figs 1, 2, 3*). Our specimens closely match the illustrations of the gonopods of the type specimen of *Scaphiostreptus parilis acuticonus* Attems 1914 (Figs 4, 5, 6), but do not exhibit the elongated prefemoral process of the first leg pair of the males as illustrated in the type specimens of *S. fischeri*; *Spiropoeus lingulatus* Karsch, 1881; and *Mardonius piceus* Attems, 1952 (Hoffman et al. 2001). The names *S. parilis acuticonus* and *S. lingulatus* and six others scattered throughout West Africa are junior subjective synonyms of *S. fischeri* (Hoffman et al. 2001). This suggests a degree of morphological variation throughout the geographic distribution of the species or a complex of closely-related species.

*Spiropoeus fischeri* is known from several localities across West Africa, with most verifiable records from Cameroon (Attems 1914, Attems 1952, Nzoko Fiemapong 2023). Although the type locality of *S. fischeri* is unknown, historical synonyms were described, based on material from Cameroon and other West African countries like Liberia and Benin (Karsch 1881, Porat 1888, Attems 1914, Brölemann 1926, Attems 1952). The type locality of Spirostreptus (Nodopyge) lingulatus Karsch, 1881 is “Congo” without further data (Karsch 1881), while the type locality of *Scaphiostreptus parilis acuticonus* Attems, 1914 is (in Attems’s handwriting) “*N’Yong, Kamerun*”, which refers to various locations in the Nyong River region, including Nyong-et-Kéllé, Nyong-et-So'o and Nyong-et-Mfoumou departments. This suggests that the species has a broader geographical distribution that spans the Guineo-Congolian rainforest region.

### Conclusion

We found that the millipede *Spiropoeus fischeri* is commonly raised in zoos and kept alive for exhibits and outreach. Through a detailed examination of morphology and natural history specimens, we provide an accurate determination of material from captive bred collections. This species, together with *A. gigas* and other spirostreptids of similar appearance, are collectively known as giant African millipedes in the pet trade and in zoo collections. Although *S. fischeri* appears to be one commonly bred species, it is likely that several other species of Spirostreptidae remain unidentified in zoos. Educational collections and zoological institutions can now provide the public with accurate information about *S. fischeri*, including its scientific name as well as detailed biological insights such as its life history and home range in sub-Saharan Africa.

## Supplementary Material

XML Treatment for Spiropoeus fischeri

## References

[B14137881] Attems C. M. T. G. von (1914). Afrikanische Spirostreptiden nebst Überblick über die Spirostreptiden orbis terrarum. Zoologica (Stuttgart).

[B14137892] Attems C. M. T. G. von (1950). Über Spirostreptiden (Diplopoda). Annalen des Naturhistorischen Museums in Wien.

[B14137903] Attems C. M. T. G. von (1952). Sur trois Spirostreptides et un Polydesmide nouveaux (Myriapodes, Diplopodes) de la Côte d’Ivoire et du Cameroun. Bulletin du Muséum national d’histoire naturelle, 2e série.

[B14149909] Bond J. E., Beamer D. A., Hedin M. C., Sierwald P. (2003). Gradual evolution of male genitalia in a sibling species complex of millipedes (Diplopoda: Spirobolida: Rhinocricidae: *Anadenobolus*). Invertebrate Systematics.

[B14155728] Brandt J. F. (1833). Tentaminum quorundam monographicorum Insecta
Myriapoda Chilognathi Latreillii spectantium prodromus. Bulletin de la Societé Impériale des Naturalistes de Moscou.

[B14137923] Brandt J. F. (1841). Generis Juli specierum enumeratio, adjectis plurium, quae hucusque nondum innotuerunt specierum brevibus descriptionibus ad Musei Academiae Scientifiarum Petropolitanae specimina factis. Bulletin Scientifique publié par l’Académie Impériale des Sciences de Saint-Pétersbourg.

[B14137932] Brölemann H. W. (1926). Myriapodes recueillis en Afrique occidentale française par M. l’administrateur en chef L. Duboscq. Archives de zoologie expérimentale et générale.

[B14137941] Crawford C. S., Goldenberg S., Warburg M. R. (1986). Seasonal water balance in *Archispirostreptus
syriacus* (Diplopoda: Spirostreptidae) from mesic and xeric Mediterranean environments. Journal of Arid Environments.

[B14137950] Demange J. -M. (1964). Les appendices postériqueurs (9e paire) du diplopsegment gonopodial (VIIe) des Spirostreptoidea (Myriapodes Diplopodes). Bulletin du Muséum National d’Histoire Naturelle, 2e série.

[B14137959] Demange J. -M. (1970). Éléments d’une révision des Spirostreptidae. I. Étude de quelques caractères taxonomiques des Spirostreptinae. Bulletin de l’Institut Fondamental d’Afrique Noire, Série A.

[B14137968] Demange J. -M. (1971). Myriapodes Diplopodes récoltés au Togo, au Dahomey et en Côte d’Ivoire par M. A. Aouti. Bulletin de l’Institut Fondamental d’Afrique Noire, Série A.

[B14137977] Demange J. - M., Mauriès J. - P. (1975). Myriapodes - Diplopodes des Monts Nimba et Tonkoui (Cote d’Ivoire, Guinée) récoltés par M. Lamotte et ses collaborateurs de 1942 à 1961. Annalen, Koninklijk Museum voor Midden-Afrika - Zoologische wetenschappen.

[B14137986] Enghoff H. (2017). A new East African genus of spirostreptid millipedes (Diplopoda, Spirostreptida, Spirostreptidae), with notes on their fungal ectoparasite *Rickia
gigas*. Zootaxa.

[B14137995] Enghoff H. (2023). A new distinct, disjunct giant millipede of the genus *Spirostreptus* Brandt, 1833, from Tanzania, and a solution for orphaned *Spirostreptus* species (Diplopoda, Spirostreptida, Spirostreptidae). Zootaxa.

[B14138004] Enghoff H., Ngute A. S. K., Kwezaura R. L., Laizzer R. L., Lyatuu H. M., Mhagawale W., Mnendendo H. R., Marshall A. R. (2024). A mountain of millipedes XI. The trachystreptoform spirostreptids of the Udzungwa Mountains, Tanzania (Diplopoda, Spirostreptida, Spirostreptidae). European Journal of Taxonomy.

[B14138017] Gervais P. (1837). Études pour servir á l’histoire naturelle des Myriapodes. Annales des Sciences naturelles, Zoologie.

[B14138026] Golovatch S. I. (1997). On the identity of some millipede species described by C. O. von Porat in 1888 (Diplopoda: Spirostreptida, Spirobolida). Bulletin de l’Institut Royal des Sciences Naturelles de Belgique, Entomologie.

[B14138035] Golovatch S. I., Hoffman R. L. (2000). On the diplopod taxa and type material of J. F. Brandt, with some new descriptions and identities (Diplopoda). Fragmenta faunistica, Supplement.

[B14138044] Hoffman R. L., Golovatch S. I., Hamer M. (2001). Identities of the millipede genera *Spirostreptus* Brandt, 1833 and Spiropoeus Brandt, 1833 (Diplopoda, Spirostreptida, Spirostreptidae). Myriapodologica.

[B14138062] Hopkin S. P., Read H. J. (1992). The Biology of Millipedes.

[B14138078] Karsch F. (1881). Neue Juliden des Berliner Museums, als Prodromus einer Juliden-Monographie. Zeitschrift für die gesammten Naturwissenschaften.

[B14138087] Krabbe E. (1982). Systematik der Spirostreptidae (Diplopoda, Spirostreptomorpha). Abhandlungen und Verhandlungen des Naturwissenschaftlichen Vereins in Hamburg (N.F.).

[B14149880] Means J. C., Francis E. A., Lane A. A., Marek P. E. (2015). A general methodology for collecting and preserving xystodesmid and other large millipedes for biodiversity research. Biodiversity Data Journal.

[B14138096] Means J. C., Marek P. E. (2017). Is geography an accurate predictor of evolutionary history in the millipede family Xystodesmidae?. PeerJ.

[B14138105] Mwabvu T., Hamer M., Slotow R., Barraclough D. (2010). A revision of the taxonomy and distribution of *Archispirostreptus* Silvestri 1895 (Diplopoda, Spirostreptida, Spirostreptidae), and description of a new spirostreptid genus with three new species. Zootaxa.

[B14149948] Nzoko Fiemapong A. R. (2023). Project N° 36690-B: Conservation of the Giant African Millipede Community in the Cameroon’s Rain-Forest Zones: distribution, threats and research needs. Final Report. https://www.rufford.org/projects/nzoko-fiemapong-armand-richard/conservation-of-the-giant-african-millipede-community-in-the-cameroons-rain-forest-zones-distribution-threats-and-research-needs/.

[B14138114] Pimvichai P., Enghoff H., Panha S. (2010). The Rhynchoproctinae, a south-east Asiatic subfamily of giant millipedes: cladistic analysis, classification, four new genera and a deviating new species from north-west Thailand (Diplopoda: Spirostreptida: Harpagophoridae). Invertebrate Systematics.

[B14138123] Porat C. O. von (1888). Über einige exotische Iuliden des Brüsseler-Museums. Annales de la Société Entomologique de Belgique.

[B14270532] Sigling S. (2010). Professional Breeders Series: Millipedes.

